# A *GRX1* Promoter Variant Confers Constitutive Noisy Bimodal Expression That Increases Oxidative Stress Resistance in Yeast

**DOI:** 10.3389/fmicb.2018.02158

**Published:** 2018-09-19

**Authors:** Jian Liu, Delphine Lestrade, Sevan Arabaciyan, Julien Cescut, Jean-Marie François, Jean-Pascal Capp

**Affiliations:** ^1^Laboratoire d’Ingénierie des Systèmes Biologiques et des Procédés, UMR CNRS 5504, UMR INRA 792, Institut National des Sciences Appliquées de Toulouse, Université de Toulouse, Toulouse, France; ^2^Toulouse White Biotechnology, UMS INRA 1337, UMS CNRS 3582, Institut National des Sciences Appliquées de Toulouse, Toulouse, France

**Keywords:** stochastic gene expression, *Saccharomyces cerevisiae*, single-cell analysis, bimodal expression, noise, phenotypic heterogeneity

## Abstract

Higher noise in the expression of stress-related genes was previously shown to confer better resistance in selective conditions. Thus, evolving the promoter of such genes toward higher transcriptional noise appears to be an attractive strategy to engineer microbial strains with enhanced stress resistance. Here we generated hundreds of promoter variants of the *GRX1* gene involved in oxidative stress resistance in *Saccharomyces cerevisiae* and created a yeast library by replacing the native *GRX1* promoter by these variants at the native locus. An outlier clone with very strong increase in noise (6-times) at the same mean expression level as the native strain was identified whereas the other noisiest clones were only 3-times increased. This variant provides constitutive bimodal expression and consists in 3 repeated but differently mutated copies of the *GRX1* promoter. In spite of the multi-factorial oxidative stress-response in yeast, replacement of the native promoter by this variant is sufficient alone to confer strongly enhanced resistance to H_2_O_2_ and cumene hydroperoxide. New replacement of this variant by the native promoter in the resistant strain suppresses the resistance. This work shows that increasing noise of target genes in a relevant strategy to engineer microbial strains toward better stress resistance. Multiple promoter replacement could synergize the effect observed here with the sole *GRX1* promoter replacement. Finally this work suggests that combining several mutated copies of the target promoter could allow enhancing transcriptional-mediated noise at higher levels than mutating a single copy by providing constitutive bimodal and highly heterogeneous expression distribution.

## Introduction

The stochastic nature of the chemical reactions governing gene expression and the small number of molecules involved in this process create cell-to-cell variations in the mRNA and protein contents (McAdams and Arkin, 1999), even among clonal cells and in homogeneous environments. This phenomenon called gene expression noise affects all genes but the level of noise depends on the gene function, suggesting a positive selection during evolution (Newman et al., 2006). For instance, noise minimization arises for genes coding for housekeeping proteins and complex-forming proteins (Fraser et al., 2004) while genes involve in stress response exhibit higher levels of noise (Newman et al., 2006).

The first modern studies of gene expression noise in bacteria and yeast (Elowitz et al., 2002; Blake et al., 2003; Raser and O’Shea, 2004) were rapidly followed by experimental evidences of its adaptive role in stressful environments or during nutritional shift (Blake et al., 2006; Smith et al., 2007; Acar et al., 2008; Fraser and Kaern, 2009; Ito et al., 2009; Lidstrom and Konopka, 2010; Ackermann, 2013; New et al., 2014; Venturelli et al., 2015; Wang et al., 2015). Since the pioneering work from Blake et al. (2006), several cases of stress resistance linked to phenotypic heterogeneity conferred by expression noise have been described in *Saccharomyces cerevisiae* (Smith et al., 2007; Rotem et al., 2010; Liu et al., 2015). It is assumed that this represents a bet-hedging strategy which allows subpopulations to be “pre-adapted” to variable selective conditions.

As modulation of noise for stress-related genes appears to modify the ability of a population to adapt, whether gene expression noise is under selection or not has been questioned (Richard and Yvert, 2014). Also stress-related genes and genes coding for trans-membrane transporters are expressed with higher noise than other genes (Bar-Even et al., 2006; Newman et al., 2006; Zhang et al., 2009; Lehner, 2010), suggesting that this phenomenon contributes to adaptation to changing environments (Liu et al., 2016). On the contrary, housekeeping and essential genes that are supposed to require stable expression to provide stable properties are expressed with lower noise (Newman et al., 2006; Lehner, 2008). Finally, gene order and chromosome organization seem to be highly linked to reduction of noise: for instance, clustering of essential genes in the genome seems to indicate negative selection on noise (Batada and Hurst, 2007), suggesting again that the level of noise is under selection and that reduction or increase of noise can be acquired by various genetic and epigenetic ways.

Many genetic determinants in promoters play a role in the generation of noise in eucaryotes (Sanchez et al., 2013), especially transcription factor (TF) binding sites (Murphy et al., 2007; Octavio et al., 2009; To and Maheshri, 2010; Sharon et al., 2014) and TATA box (Raser and O’Shea, 2004; Blake et al., 2006; Hornung et al., 2012). Also, promoter with nucleosome binding sites harbors ON vs. OFF alternative states and thus produces bursts of mRNA production which generate cell-to-cell variability in gene expression (Sanchez and Golding, 2013). Promoters containing poly-nucleosome-disfavoring sequences have lower noise due to higher transcription burst frequency (Sharon et al., 2014). The impact of these genetic determinants on noise has been mostly studied by several works aiming at modifying promoter sequences either rationally or randomly (Murphy et al., 2007, 2010; Hornung et al., 2012; Carey et al., 2013; Sharon et al., 2014). These strategies helped to decipher the origin of noise but they have never been used to generate promoter variants of stress-related genes that might enhance resistance to specific stresses by producing higher noise in their expression.

In yeast, oxidative stress is one of the most studied because of its importance in many basic (aging) and industrial processes. Especially, reactive oxygen species (ROS) are generated by various mechanisms in bioprocessing using yeast and can limit the efficiency of its use in biotechnological applications (Wiseman, 2005; Fu et al., 2014; Landi et al., 2015). It is also one of the most complex stresses with 37 protective enzymes that are up-regulated in response to ROS exposure in yeast (Morano et al., 2012). Among this family, the glutathione-dependent disulfide oxidoreductases (glutaredoxins) such as Grx1 are part of the glutaredoxin system where glutaredoxins are oxidized by substrates, and reduced non-enzymatically by glutathione (Luikenhuis et al., 1998; Collinson et al., 2002; Collinson and Grant, 2003). Especially, elevated gene dosage of *GRX1* confers resistance to peroxides including hydrogen peroxide (H_2_O_2_), tert-butyl hydroperoxide and cumene hydroperoxide in yeast (Collinson et al., 2002). This suggests that increasing expression noise of this gene would produce a higher resistant subpopulation with increased Grx1 levels that could favor survival and growth upon challenging the yeast culture with an oxidative stress.

Thus the purpose of this work was to evaluate the hypothesis that resistance of yeast strains to oxidative stress could be improved by modifying the expression variability of *GRX1* without changing its mean expression. To this end, we generated hundreds of *S. cerevisiae* *GRX1* promoter variants and identified an evolved yeast clone that harbors a candidate variant that strongly enhances noise without changing the mean expression level and which was associated with increased resistance to H_2_O_2_ and cumene hydroperoxide. We furthermore found that this promoter variant conferred a bimodal expression profile that was lost upon replacement with the native promoter in the evolved strain. Altogether, this work shows that varying the noise level in the expression of a gene implicated a given stress could be alternative strategy to readily isolate evolved population with higher resistance to this given stress.

## Materials and Methods

### Yeast Strains and Growth Conditions

The yeast strain *GRX1-GFP-HIS3MX6* was purchased from Thermo Ficher Scientific. All the primers used in this study are listed in **Supplementary Table 1**. To create the *GRX1-GFP-tdTomato-kanR* strain, a PCR fragment containing tdTomato-kanR and homologies to *GFP-HIS3MX6* was amplified with primers C1 and C2 from pfa6a-tdTomato-kanR (constructed in our lab) and transformed to the *GRX1-GFP-HIS3MX6* strain.

All the strains were grown in liquid YPD medium containing 20 g/L glucose (Sigma), 10 g/L peptone (Euromedex) and 10 g/L yeast extraction (Euromedex). When needed, 20 g/L agar (Euromedex) was added to make solid plates. YNB-URA^−^ plates [20 g/L glucose (Sigma), 20 g/L agar (Euromedex), 1.71 g/L yeast nitrogen base without amino acids and nitrogen (Euromedex), 5 g/L ammonium sulfate (Sigma), and 0.77 g/L CSM-URA^−^ (Euromedex)] or 5-FOA plate [20 g/L glucose (Sigma), 20 g/L agar (Euromedex), 1.71 g/L yeast nitrogen base without amino acids and nitrogen (Euromedex), 5 g/L ammonium sulfate (Sigma), 0.79 g/L CSM-URA^−^ (Euromedex), and 1 g/L 5-FOA (Euromedex)] were used to select transformants.

### Generation of the *GRX1* Promoter Variants Library

The PCR fragment containing the *GRX1* promoter (400 bp core sequence) and its flanking sequence (about 300 bp) was amplified from the genomic DNA of the *GRX1-GFP-HIS3MX6* strain with primers C3 and C4. This fragment was cloned into the plasmid pfa6a-GFP-kanR through EcoRI and SalI (now named pfa6a-GRX1).

The GeneMorph II Random Mutagenesis Kit (Agilent) was used to amplify the *GRX1* core promoter with random mutations from pfa6a-GRX1 with primers C5 and C6 following the standard protocol of the manufacturer (a total of 50 cycles of amplification was applied). The purified PCR product was used as primers to amplify the rest of the plasmids by the Phusion DNA polymerase (NEB). The final product was digested by DpnI, purified, and then transformed to DH5α (NEB) following the standard protocol of the manufacturer (a total of 10^5^ transformants were obtained and pooled together). 14 transformants were isolated and sequenced to estimate mutation frequencies.

The *GRX1* promoter in the *GRX1-GFP-tdTomato-kanR* strain was first replaced by a PCR fragment containing *URA3* which was amplified from the plasmid pJRL2-TATA-CFP (Addgene) with primers C7 and C8. Then *URA3* was replaced by the fragments of pfa6a-GRX1 containing a variant promoter which was cut from the pooled plasmids by EcoRI and SalI through 5-FOA selection (a total of 10^5^ transformants were obtained and pooled together).

### Screening of Single Clones Containing *GRX1* Promoter Variants and Cell Sorting

Single cells from the library were isolated by the MoFlo Astrios EQ cell sorter with the Summit v6.3 software (Beckman Coulter, Brea, CA, United States). Cultures at stationary phase were diluted 20 times and grown at 30°C with vigorous shaking (200 rpm) for 6 h before cell sorting (final OD≈2). Cultures were spun down at 3,000 *g* for 5 min at 4°C. Growth media was removed, and cells were re-suspended in ice-cold PBS. The SmartSampler and microplate holder were kept at 4°C during cell sorting. Cell sorting was carried out with a 70 μm nozzle and 60 psi operating pressure. The sorting speed was kept at around 30,000 events per second. The single mode for the sort mode and 0.5 drop for the droplet envelope were chosen. Based on the FSC-Area vs. SSC-Area (488 nm laser) plot and the FSC-Height vs. FSC-Area (488 nm laser) plot, single cells with similar cell size and granularity were first selected. Then based on the histogram of the GFP-tdTomato fluorescence (560 nm laser, 614/20 filter), single cells of which the fluorescence lied in the middle 50% of the population were sorted to single wells of a 96 well plate with 200 μL YPD per well. Finally, a total of 4 plates were obtained.

The plates were kept in an incubator (30 h, 700 rpm, and 70% humidity) for 48 h till stationary phase. They were diluted 20 times and grown to exponential phase (6 h) to measure the expression profile of *GRX1-GFP-tdTomao* of each clone by MACSQuant^®^ VYB with the MACSQuantify^TM^ Software (Miltenyi Biotec, Germany). A total of 10^5^ cells were analyzed for each clone, and the fsc files were exported and analyzed by R program (v3.2) with the Bioconductor packages (v3.0). A norm2Filter filter was applied on FSC-A/SSC-A to select homogeneous cells regarding size, shape, and cellular complexity. The GFP-tdTomato fluorescence (Channel Y2-A) was transformed with the log function. Then the mean GRX1-GFP-tdTomato fluorescence value and its noise (the square of the coefficient of variance) was calculated and exported. All the figures were drawn based on the transformed data. Experiments were repeated three times.

To analyze the dynamics of bimodality recovering in the bimodal clone, the BY4741 strain was used as a negative (non-fluorescent) control and for calibrations. Both strains were grown overnight at 30°C in YPD medium and diluted 10 times in the morning. After 6 h, 10^6^ cells around each peak in the bimodal clone were sorted simultaneously with MoFlo Astrios EQ (Beckman Coulter, Brea, CA, United States). The unimodality of the sorted cells was checked and they were then grown at 30°C in YPD medium. The recovery of bimodality was followed with MACSQuant VYB (Miltenyi Biotec, Germany).

### Promoter Swapping

The *GRX1* promoter variant of the outlier clone was first replaced by *URA3* as described above and then replaced by the native promoter cloned by PCR. To study each mutated copy identified in the *GRX1* promoter variant of the outlier clone, the core promoter of *GRX1* from this outlier was amplified by PCR with primers C5 and C6. The purified PCR product was used as primers to amplify the plasmid pfa6a-GRX1. The final product was digested by DpnI, purified and transformed to DH5α. The plasmids extracted from 10 clones were sequenced. Each of the three mutated copies was identified as a single copy in different clones. Then the promoters with a single mutated core sequence were cut from the plasmid and replaced the *URA3* fragment as described above. The expression profile of all these strains was verified by MACSQuant^®^ VYB with the MACSQuantify^TM^ Software, and all the data were analyzed by R program as described above. Experiments were repeated three times.

### Resistance to the Oxidative Reagents

Overnight cultures were diluted to *OD* = 0.2 and cells grown in YPD medium until late exponential phase (7 h). Then they were diluted 100-times in YPD cultures with different concentrations of oxidative reagents [H_2_O_2_ (Sigma, 0–9 mM) or cumene hydroperoxide (Sigma, 0–160 μM)]. The OD of each culture was measured after 30 h. The residual growth was calculated as the OD with a specific concentration of one reagent divided by the OD in YPD. For experimental growth time course in 5.5 mM H_2_O_2_ or without H_2_O_2_, OD was followed during 35 h to draw the growth curve. All these experiments were repeated at least three times.

For the spot assays, overnight cultures were diluted to *OD* = 0.2 and grown in YPD medium until late exponential phase (7 h). All the final cultures were adjusted to OD 1, then 10 μL of each culture was dropped on YPD plates containing either 4.5 mM H_2_O_2_, 2 mM diamide, 2 mM tert-butyl hydroperoxide, or 1 g/L furfural. All the plate were kept at 30 h for 2 days. All these experiments were repeated at least three times.

## Results

### Random Mutagenesis of the *GRX1* Promoter and Library Construction

The *GRX1* gene was chosen to evaluate our hypothesis because it is one of the most expressed oxidative-stress response genes in non-selective conditions (Newman et al., 2006), and hence this should facilitate measurement of its expression noise level using flow cytometry experiments. However, we found that the fluorescence conferred by a fusion protein Grx1-GFP (Newman et al., 2006) was not satisfactorily distinct from the fluorescence background of a yeast population that do not bear this fusion protein (**Supplementary Figure 1**). Therefore, we decided to create a double fluorescent marker with tdTomato added in C-terminal which allowed us to obtain a distribution that does not overlap the control population (**Supplementary Figure 1**).

**FIGURE 1:**
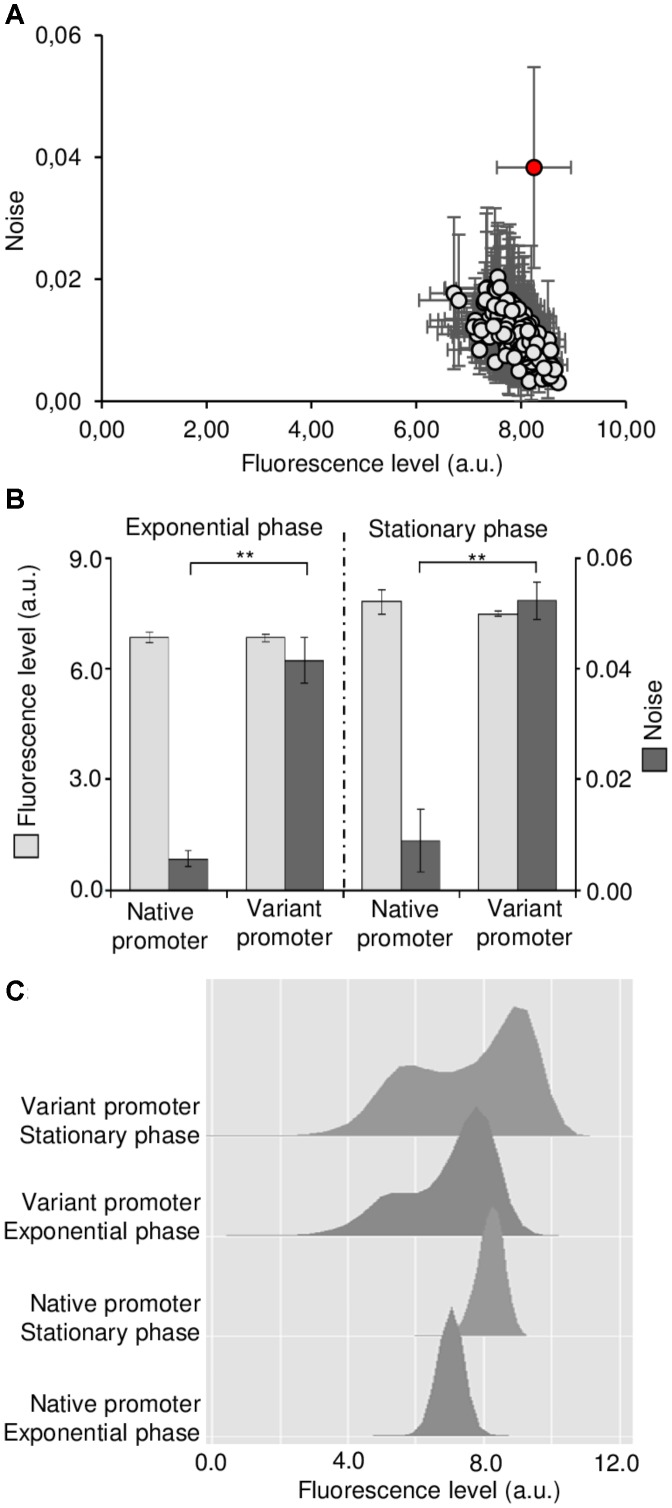
Identification of a *GRX1* promoter variant conferring constitutive bimodal expression profile. **(A)** Mean expression and noise levels of about 200 *S. cerevisiae* clones each containing a variant version of the *GRX1* promoter (*pGRX1*) at its original locus. The outlier clone with the highest noise is highlighted in red. Results are the mean of three independent experiments with standard deviation. **(B)** Mean expression (light gray) and noise (dark gray) levels conferred by the native and the noisiest *pGRX1* variant in exponential (left) or stationary (right) phase. Results are the mean of three independent experiments with standard deviation. A significant statistical difference is represented by (^∗∗^) when *p* < 0.01 in *T*-test. **(C)** Expression profiles conferred by the native and the noisiest variant *pGRX1* in exponential or stationary phase.

We then considered that we had the necessary brightness to evaluate the effects of *GRX1* promoter mutations in a wide range of expression distribution. These variants were obtained after three rounds of error-prone PCR on the 400 bp *GRX1* promoter. Sequencing of 14 clones showed that 2.73 ± 1.49 mutations were present per promoter with “G–A” and “C–T” representing about half of the mutations (frequencies are given in the **Supplementary Table 2**). Then we first created a library in *Escherichia coli* before integrating it in replacement of the native promoter in the *S. cerevisiae* laboratory strain BY4741. The strategy consisted in replacing the native promoter by the auxotrophic marker *URA3*, and then replacing *URA3* by the variants using the classical recombination procedure in yeast. We finally obtained a library *S. cerevisiae* clones, with each of them containing potentially a mutated version of the *GRX1* promoter. Clones were distributed in 96-well plates and analyzed by high-throughput flow cytometry to measure their mean and noise levels (**Supplementary Table 3**). We found the expected correlation where decreased mean is associated to increased noise, but very interestingly, we identified an outlier clone that exhibited a noise level approximatively 2-times higher than the second noisiest clone (0.0383 vs. 0.0204) (**Figure 1A**).

### Sequencing and Expression Profile of the *GRX1* Promoter Variant With the Highest Noise

This outlier clone was further analyzed in comparison with the native promoter in exponential and stationary phase (**Figure 1B**). In both phases, its mean expression level was the same as the one conferred by the native promoter, but its noise level was increased about 6-times (*p* < 0.01). The mean expression levels were increased in stationary phase, associated with an increased noise for the mutated promoter. Moreover, when examining the expression profiles by flow cytometry, a bimodal distribution was clearly visible both exponential and stationary phase while the native promoter conferred unimodal expression (**Figure 1C**). The peak corresponding to the low-expressers is slightly above the fluorescence background, showing that expression is weak but high enough to be above the threshold of detection in this subpopulation (**Supplementary Figure 2**). The slight increase in mean expression in stationary phase is noticeable for both the native and the mutated promoters, but for the latter only the peak with the highest expression moved to higher expression levels. This logically led to increased noise in stationary phase because the population as a whole becomes even more heterogeneous. We also followed the dynamics of recovering of the bimodal profile after cell sorting of either the low-expressing cells or the high-expressing cells. Growth of these subpopulations in non-selective media showed that bimodality was restored after 16 h in both cases (**Supplementary Figure 3**), showing that the two states are highly reversible and epigenetic in nature, and that and the switching rate seems to be the same for both subpopulations.

**FIGURE 2:**
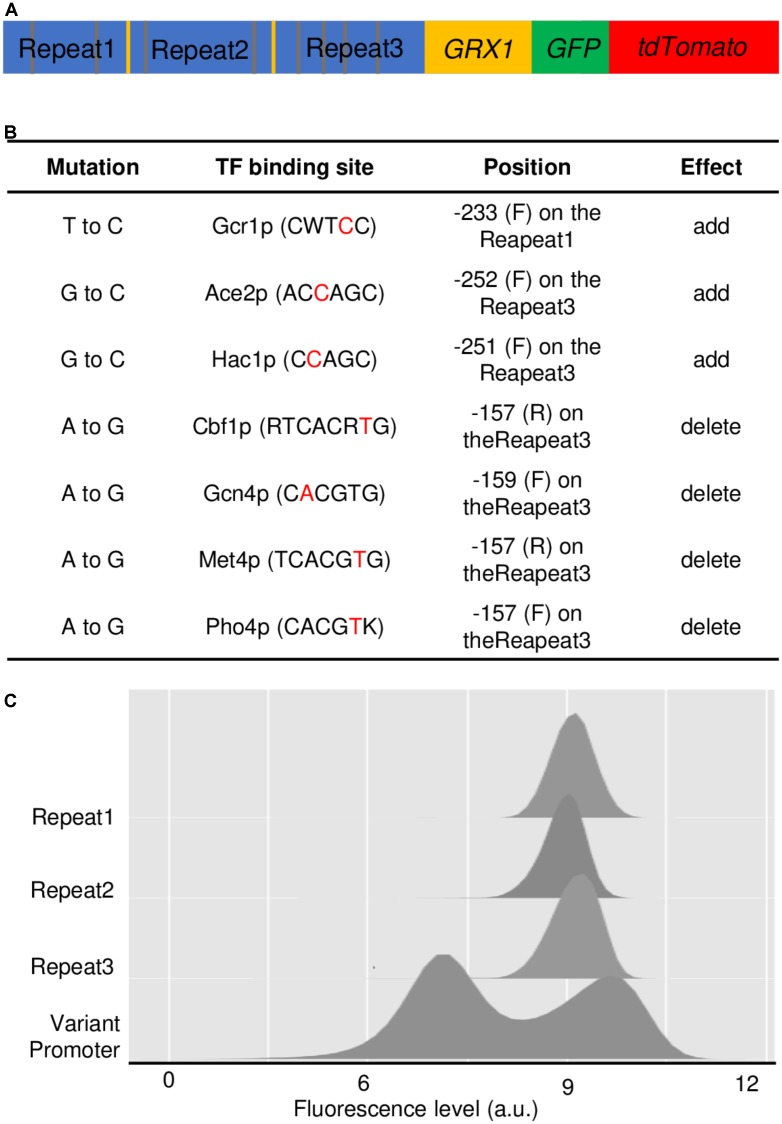
Characterization of the noisiest *GRX1* promoter variant. **(A)** Schematic representation of the noisiest variant *pGRX1* variant and the downstream encoded gene: three repeated but differently mutated copies of *pGRX1* (blue) separated by few codons of the beginning of the *GRX1* ORF (yellow), the *GRX1* (yellow), GFP (green), and tdTomato (red) ORF. **(B)** Effects of the mutations in *pGRX1* that add or delete transcription factor (TF) binding sites. For each mutation (first column), the consensus TF binding site is given (second column) with the site of the mutation highlighted in red, the position of the mutation, the modified repeat and the modified strand (F for Forward, R for Reverse) (third column) and the effect of the mutation on the TF binding site (addition or deletion) (fourth column). **(C)** Expression profiles conferred by the noisiest variant *pGRX1* and each of the mutated repeat that constitutes this variant *pGRX1*.

Sequencing of the mutated promoter revealed an expected structure with three successive repeats of the *GRX1* each mutated differently (**Figure 2A**, see **Supplementary Figure 4** for the full sequence). These copies were numbered repeat 1, 2, and 3 from 5’ to 3’. Repeat 1, 2, and 3 contained two mutations, one mutation and one deletion, and four mutations, respectively. Some of these mutations suppress or create TF binding sites, mainly in repeat 3 (**Figure 2B**). Moreover, few codons of the beginning of the ORF are present at the junction between repeats 1 and 2, and between repeats 2 and 3, and correspond to the sequence of the reverse primer initially used to amplify the promoter sequence (**Supplementary Figure 4**). Moreover 7 nucleotides were added at the junctions, suggesting that this extremely rare junctional event occurred in the cell during the process of library construction and transformation.

To decipher whether one or two or three repeats were needed to confer bimodality, we cloned each repeat independently to replace the native promoter. None of the three copies was able to confer bimodality by its own (**Figure 2C**). They all provided very similar unimodal expression distribution, showing that only the combinatorial action of several repeats is able to modify the expression profile. Interestingly, despite the relatively homogenous distribution conferred by each repeat independently, the bimodal distribution given by the three repeats cover a wide range of expression levels, from cells in the auto-fluorescence background to cells expressing *GRX1* at far higher levels than the highest levels given by a unique repeat.

### The Noisiest *GRX1* Promoter Variant Improves H_2_O_2_ and Cumene Hydroperoxide Resistance

**FIGURE 3:**
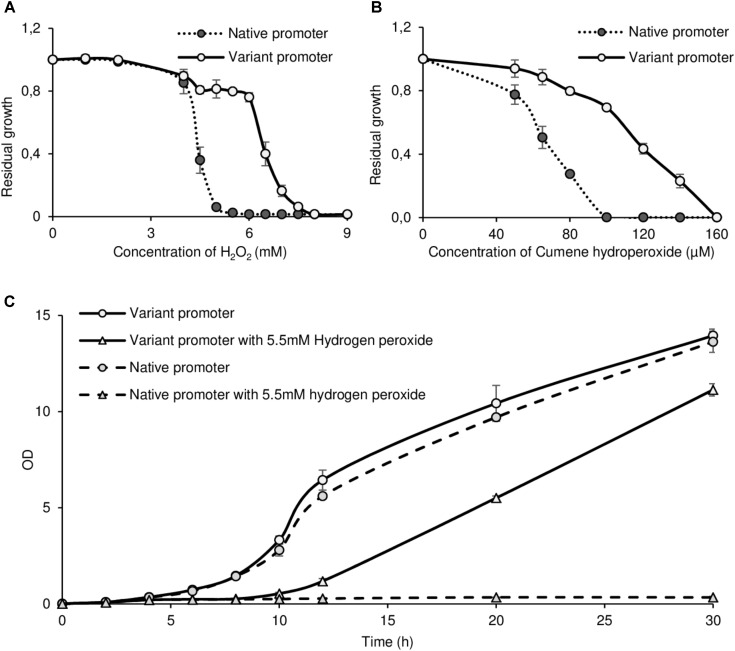
The constitutive bimodal expression pattern produced by the noisiest *GRX1* promoter variant confers oxidative stress resistance. **(A,B)** Residual growth of strains containing either the native or the noisiest *pGRX1* variant in rich medium after 30 h in increasing H_2_O_2_ **(A)** or cumene hydroperoxide **(B)** concentrations. Residual is defined as the OD after 30 h in non-selective rich medium divided by the OD after 30 h in rich medium containing H_2_O_2_ or cumene hydroperoxide. **(C)** Example of growth curves of strains containing either the native or the noisiest *pGRX1* variant in rich medium containing or not 5.5 mM H_2_O_2_. All results are the mean of three independent experiments with standard deviation.

Given our initial hypothesis, we tested a possible increase in oxidative stress resistance in this strain. As *GRX1* overexpression confers resistance to peroxides in yeast (Collinson et al., 2002), we grew the control strain containing the native promoter and the strain containing the mutated promoter in a wide range of hydrogen peroxide (H_2_O_2_) and cumene hydroperoxide concentrations. These compounds represent typical inorganic and organic peroxides, respectively. When considering the residual growth in each concentration (OD after 30 h in peroxide-containing medium divided by OD after 30 h in control medium), a strong increase in resistance is observed for both compounds: from 4.5 to 7 mM H_2_O_2_ (**Figure 3A**) and from 50 to 140 μM cumene hydroperoxide (**Figure 3B**).

A representative growth curve in H_2_O_2_ shows that while both strains grew similarly in the non-selective medium, only the mutated promoter allowed growth in 5.5 mM H_2_O_2_ (**Figure 3C**). The same phenomenon is observed from 5 to 7 mM H_2_O_2_ and from 100 to 140 μM cumene hydroperoxide where no growth was observed with the native promoter. Another example of growth curves without or with 100 μM cumene hydroperoxide in given in **Supplementary Figure 5**. Spot assays were also performed with 4.5 mM H_2_O_2_ in rich medium and showed better growth with the mutated promoter both with cells in exponential and in stationary phase (**Supplementary Figure 6**), confirming the increased resistance observed in liquid medium. Other compounds producing oxidative stress response were tested in spot assays: diamide 2 mM, tert-butyl hydroperoxide 2 mM, and furfural 1 g/L where the strains did not show any difference (**Supplementary Figure 6**).

### Bimodality and Oxidative Stress Resistance Is Specifically Conferred by This Promoter Variant of *GRX1*

**FIGURE 4:**
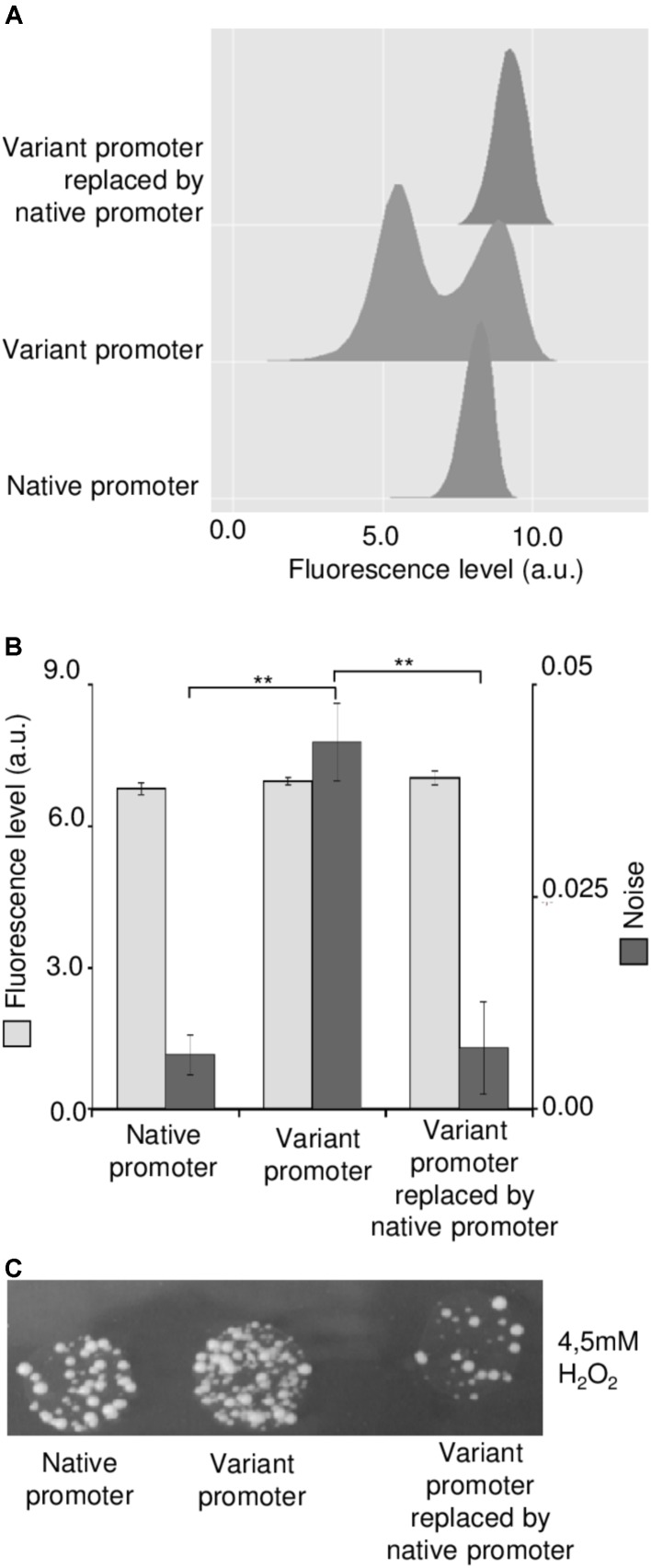
New replacement of the *pGRX1* variant in the resistant strain by the native promoter suppresses bimodality and oxidative stress resistance. **(A)** Expression profiles conferred by the native *pGRX1*, the noisiest *pGRX1* variant or the native *pGRX1* after replacement of noisiest *pGRX1* variant in the resistant strain. **(B)** Mean expression (light gray) and noise (dark gray) levels conferred by the native *pGRX1*, the noisiest *pGRX1* variant, or the native *pGRX1* after replacement of noisiest *pGRX1* variant in the resistant strain. Results are the mean of three independent experiments with standard deviation. A significant statistical difference is represented by (^∗∗^) when *p* < 0.01 in *T*-test. **(C)** Spot assay in 4.5 mM H_2_O_2_ with strains containing the native *pGRX1*, the noisiest *pGRX1* variant or the native *pGRX1* after replacement of noisiest *pGRX1* variant in the resistant strain.

To go further in deciphering the origins of the increased *GRX1* expression variability by distinguishing *cis*- and possible *trans*-effects (in other words to check that it is only due to the mutated promoter or to the combined effect of mutations in *cis* and *trans*), we decided to replace the promoter variant in the resistant strain by the native one. As shown in **Figure 4A**, we found that the bimodal expression was only produced by the mutated promoter since the expression profile returned to unimodality with the native promoter. In addition, the noise level decreased at the level of the original strain while the mean expression level was very slightly increased after swapping in the resistant strain (**Figure 4B**). This promoter swapping was also associated with the suppression of the resistance of the strain to H_2_O_2_ (**Figure 4C**).

## Discussion

Several works showed an increased stress resistance conferred by higher expression noise in *Saccharomyces cerevisiae* (Smith et al., 2007; Rotem et al., 2010; Liu et al., 2015). Especially, we previously observed that the increased expression variability conferred by a natural yeast promoter variant isolated from an oenological strain provided a clear benefit in the face of an environmental stress compared to its lab strain counterpart (Liu et al., 2015). This modulation of gene expression noise was partly due to promoter modifications. As other works revealed the possibility to get a wide range of mean and/or noise levels by random mutagenesis of promoter sequences (Hornung et al., 2012), we hypothesized that such mutagenesis of promoters related to stress-response should allow identification of interesting variants that could improve stress resistance. More precisely, this work aimed at obtaining promoters variants of the oxidative stress-related gene *GRX1* in *Saccharomyces cerevisiae* that confer higher noise at equal mean and testing the phenotypic consequences in terms of oxidative stress resistance.

We succeeded in obtaining a strain with enhanced oxidative stress resistance by this targeted (gene-specific) approach. This approach is original for stress resistance improvement because it targets specific genes while classical evolutionary engineering protocols do not. It allows better control of (and knowledge on) the intracellular events leading to increased resistance, even for multi-factorial stress responses. Moreover, contrary to other controlled strategies such as gene overexpression, modifying the expression profile of target genes without modifying their expression mean allows that there is no consumption and mobilization of energy resources to overexpress this gene when it is useless (which could be deleterious to the strain behavior in non-selective conditions). Finally, higher gene expression variability allows the pre-existence of a subpopulations with these higher levels of expression that are “pre-adapted” to the onset of stress, and cells to reach these higher levels more quickly when stress appears, which would limit cell death.

Previous works aiming at mutating target promoters to study mean–noise relationships did not identify outlier mutants in this relationship (Hornung et al., 2012). Here we found a clone harboring such a highly increased noise and bimodal expression profile at equal mean compared to the native promoter (×4 while our second noisiest was only 2-times increased), suggesting that this clone undergone an original genetic event. Indeed this noisiest clone contained a *GRX1* promoter structure consisting in three repeated by differently mutated copies of the promoter. In spite of the unknown process that led to this original structure, it reveals that promoter engineering that consists in combining several mutated copies of a promoter of interest could be a way to strongly increase noise, especially by producing bimodal expression, while mutating only a single copy does not allow such strong modification of the noise level.

Interestingly, we recently showed that bimodality in gene expression can be instigated by DNA context, inducing conditions and strain background from the same promoter sequence (Liu et al., 2018). In fact, many phenomena, especially gene regulatory networks topologies (To and Maheshri, 2010; Venturelli et al., 2012), cell signaling (Biggar and Crabtree, 2001; Paliwal et al., 2007; Birtwistle et al., 2012) or TFs dynamics, binding and regulation (Kelemen et al., 2010; Pelet et al., 2011), have a major impact on bimodality. Nevertheless, only few works showed *cis*-effects on bimodality. For instance, the spatial distribution of activator and repressor binding sites has been shown to influence gene expression to become monostable or bistable in yeast (Kelemen et al., 2010). The expression profile depends on the spatial distribution of the binding sites of the repressors along the DNA (Kelemen et al., 2010). Chromatin remodeling is another determinant of this bimodal expression behavior in yeast (Pelet et al., 2011). It seems that only a fraction of the population remodel chromatin to allow for efficient transcription at low stress levels. Genomic regions undergoing frequent chromatin remodeling such as subtelomeres also frequently display bimodal and stochastic gene expression in response to environmental stimuli (Halme et al., 2004; Domergue et al., 2005; Choi et al., 2008). Also, variegated expression of cell adhesion genes localized in subtelomeric domains may enhance the survival or virulence of fungal cells (Halme et al., 2004; Domergue et al., 2005). Interestingly, nucleoid-binding proteins in *Escherichia coli* were recently proposed to also contribute to regulation of bimodal expression of virulence genes leading to opposing bacterial fates (Leh et al., 2017). All these previous observations suggest that modification of the TF binding sites distribution, number and affinity together with modification of nucleosome occupancy and positioning are all responsible for the transition from unimodal to bimodal expression mode in our *GRX1* promoter variant.

Moreover, many bimodal expression profiles have been studied on artificial inducible systems where bimodality was produced in specific inducting conditions, rendering extrapolation to strain engineering rather difficult. On non-artificial systems, bimodality has been mostly studied in the context of nutritional and stress response in *S. cerevisiae*. Especially, downstream targets harbor bimodal expression only in specific ranges of stimulus concentrations in the galactose regulatory network (Acar et al., 2005; Venturelli et al., 2012) or the high-osmolarity glycerol (HOG) pathway (Pelet et al., 2011), suggesting that this expression mode could have been selected for at least in certain stressful conditions thanks to an adaptive benefit for the population through a bet-hedging strategy. Here we showed that it is possible to obtain promoter variants that constitutively drive noisy bimodal expression from a unimodal native promoter. This avoids the need for specific inducer or repressor and precise control of growth conditions, and shows that bet-hedging in a very wide range of gene expression could be counter-intuitively not detrimental in optimal growth conditions while being highly in beneficial in selective conditions. Indeed, the sole replacement of the native promoter by the variant conferring this basal bimodal profile of stress-response genes is sufficient alone to allow better resistance in stressful conditions.

While oxidative stress response involves 37 different enzymes in *S. cerevisiae*, modifying expression variability of *GRX1* alone produced phenotypic effects here, suggesting that combining the same strategy for several genes involved in the same stress response could produce additive or even synergistic effects. To engineer strains of interest toward even higher resistance, either the same variant could be placed upstream other genes involved in resistance to the same stress (here for instance the *GRX2* gene which is a paralog of *GRX1)*, or the same strategy of generating noisier promoter variants could be applied to these other genes to reach an optimal effect from their native promoters.

Compared to *in vivo* evolution methods using turbidostat or chemostat culture that can take approximately between few months and a year, the method presented here could be much more efficient and save time for strain improvement. Moreover, in these “classical” methods, a strong constraint must be defined *a priori*, while it is not needed to define here such constraints at the outset but to choose/identify a target gene linked to a given stress or to a specific metabolic function. This may seem easier especially when the constraint for *in vivo* evolution is difficult to implement. In conclusion, this work shows that varying expression noise of a gene linked to a relevant biotechnology trait such as oxidative or osmotic stress could be an alternative strategy in reverse engineering of industrial strains.

## Author Contributions

JL, J-MF, and J-PC conceived and designed the experiments. JL, DL, SA, and J-PC acquired, analyzed or interpreted the data. JL and J-PC wrote the manuscript.

## Conflict of Interest Statement

The authors declare that the research was conducted in the absence of any commercial or financial relationships that could be construed as a potential conflict of interest.

## References

[B1] Acar M. Becskei A. Van Oudenaarden A. (2005). Enhancement of cellular memory by reducing stochastic transitions. *Nature* 435 228–232. 10.1038/nature03524 15889097

[B2] Acar M. Mettetal J. T. Van Oudenaarden A. (2008). Stochastic switching as a survival strategy in fluctuating environments. *Nat. Genet.* 40 471–475. 10.1038/ng.110 18362885

[B3] Ackermann M. (2013). Microbial individuality in the natural environment. *ISME J.* 7 465–467. 10.1038/ismej.2012.131 23178672 PMC3578555

[B4] Bar-Even A. Paulsson J. Maheshri N. Carmi M. O’Shea E. Pilpel Y. (2006). Noise in protein expression scales with natural protein abundance. *Nat. Genet.* 38 636–643. 10.1038/ng1807 16715097

[B5] Batada N. N. Hurst L. D. (2007). Evolution of chromosome organization driven by selection for reduced gene expression noise. *Nat. Genet.* 39 945–949. 10.1038/ng2071 17660811

[B6] Biggar S. R. Crabtree G. R. (2001). Cell signaling can direct either binary or graded transcriptional responses. *EMBO J.* 20 3167–3176. 10.1093/emboj/20.12.3167 11406593 PMC150188

[B7] Birtwistle M. R. Rauch J. Kiyatkin A. Aksamitiene E. Dobrzynski M. Hoek J. B. (2012). Emergence of bimodal cell population responses from the interplay between analog single-cell signaling and protein expression noise. *BMC Syst. Biol.* 6:109 10.1186/1752-0509-6-109 PMC3484110 22920937

[B8] Blake W. J. Balazsi G. Kohanski M. A. Isaacs F. J. Murphy K. F. Kuang Y. (2006). Phenotypic consequences of promoter-mediated transcriptional noise. *Mol. Cell.* 24 853–865. 10.1016/j.molcel.2006.11.003 17189188

[B9] Blake W. J. KAErn M. Cantor C. R. Collins J. J. (2003). Noise in eukaryotic gene expression. *Nature* 422 633–637. 10.1038/nature01546 12687005

[B10] Carey L. B. Van Dijk D. Sloot P. M. Kaandorp J. A. Segal E. (2013). Promoter sequence determines the relationship between expression level and noise. *PLoS Biol.* 11:e1001528 10.1371/journal.pbio.1001528 PMC3614515 23565060

[B11] Choi J. K. Hwang S. Kim Y. J. (2008). Stochastic and regulatory role of chromatin silencing in genomic response to environmental changes. *PLoS One* 3:e3002 10.1371/journal.pone.0003002 PMC2500160 18714342

[B12] Collinson E. J. Grant C. M. (2003). Role of yeast glutaredoxins as glutathione S-transferases. *J. Biol. Chem.* 278 22492–22497. 10.1074/jbc.M301387200 12684511

[B13] Collinson E. J. Wheeler G. L. Garrido E. O. Avery A. M. Avery S. V. Grant C. M. (2002). The yeast glutaredoxins are active as glutathione peroxidases. *J. Biol. Chem.* 277 16712–16717. 10.1074/jbc.M111686200 11875065

[B14] Domergue R. Castano I. De Las Penas A. Zupancic M. Lockatell V. Hebel J. R. (2005). Nicotinic acid limitation regulates silencing of Candida adhesins during UTI. *Science* 308 866–870. 10.1126/science.1108640 15774723

[B15] Elowitz M. B. Levine A. J. Siggia E. D. Swain P. S. (2002). Stochastic gene expression in a single cell. *Science* 297 1183–1186. 10.1126/science.1070919 12183631

[B16] Fraser D. Kaern M. (2009). A chance at survival: gene expression noise and phenotypic diversification strategies. *Mol. Microbiol.* 71 1333–1340. 10.1111/j.1365-2958.2009.06605.x 19220745

[B17] Fraser H. B. Hirsh A. E. Giaever G. Kumm J. Eisen M. B. (2004). Noise minimization in eukaryotic gene expression. *PLoS Biol.* 2:e137 10.1371/journal.pbio.0020137 PMC400249 15124029

[B18] Fu Z. Verderame T. D. Leighton J. M. Sampey B. P. Appelbaum E. R. Patel P. S. (2014). Exometabolome analysis reveals hypoxia at the up-scaling of a *Saccharomyces cerevisiae* high-cell density fed-batch biopharmaceutical process. *Microb Cell Fact.* 13:32 10.1186/1475-2859-13-32 PMC4016033 24593159

[B19] Halme A. Bumgarner S. Styles C. Fink G. R. (2004). Genetic and epigenetic regulation of the FLO gene family generates cell-surface variation in yeast. *Cell* 116 405–415. 10.1016/S0092-8674(04)00118-7 15016375

[B20] Hornung G. Bar-Ziv R. Rosin D. Tokuriki N. Tawfik D. S. Oren M. (2012). Noise-mean relationship in mutated promoters. *Genome Res.* 22 2409–2417. 10.1101/gr.139378.112 22820945 PMC3514670

[B21] Ito Y. Toyota H. Kaneko K. Yomo T. (2009). How selection affects phenotypic fluctuation. *Mol. Syst. Biol.* 5:264 10.1038/msb.2009.23 PMC2683726 19401676

[B22] Kelemen J. Z. Ratna P. Scherrer S. Becskei A. (2010). Spatial epigenetic control of mono- and bistable gene expression. *PLoS Biol.* 8:e1000332 10.1371/journal.pbio.1000332 PMC2838748 20305717

[B23] Landi C. Paciello L. De Alteriis E. Brambilla L. Parascandola P. (2015). High cell density culture with *S.* *cerevisiae CEN.PK*113-5D for IL-1beta production: optimization, modeling, and physiological aspects. *Bioprocess Biosyst. Eng.* 38 251–261. 10.1007/s00449-014-1264-8 25106469

[B24] Leh H. Khodr A. Bouger M. C. Sclavi B. Rimsky S. Bury-Mone S. (2017). Bacterial-chromatin structural proteins regulate the bimodal expression of the locus of enterocyte effacement (LEE) pathogenicity island in Enteropathogenic *Escherichia coli*. *mBio* 8:e00773-17. 10.1128/mBio.00773-17 PMC5550750 28790204

[B25] Lehner B. (2008). Selection to minimise noise in living systems and its implications for the evolution of gene expression. *Mol. Syst. Biol.* 4:170 10.1038/msb.2008.11 PMC2290932 18319722

[B26] Lehner B. (2010). Conflict between noise and plasticity in yeast. *PLoS Genet.* 6:e1001185 10.1371/journal.pgen.1001185 PMC2973811 21079670

[B27] Lidstrom M. E. Konopka M. C. (2010). The role of physiological heterogeneity in microbial population behavior. *Nat. Chem. Biol.* 6 705–712. 10.1038/nchembio.436 20852608

[B28] Liu J. Arabaciyan S. Francois J. M. Capp J. P. (2018). Bimodality of gene expression from yeast promoter can be instigated by DNA context, inducing conditions and strain background. *FEMS Yeast Res.* 18:foy047 10.1093/femsyr/foy047 29684123

[B29] Liu J. Francois J. M. Capp J. P. (2016). Use of noise in gene expression as an experimental parameter to test phenotypic effects. *Yeast* 33 209–216. 10.1002/yea.3152 26802744

[B30] Liu J. Martin-Yken H. Bigey F. Dequin S. Francois J. M. Capp J. P. (2015). Natural yeast promoter variants reveal epistasis in the generation of transcriptional-mediated noise and its potential benefit in stressful conditions. *Genome Biol. Evol.* 7 969–984. 10.1093/gbe/evv047 25762217 PMC4419794

[B31] Luikenhuis S. Perrone G. Dawes I. W. Grant C. M. (1998). The yeast *Saccharomyces cerevisiae* contains two glutaredoxin genes that are required for protection against reactive oxygen species. *Mol. Biol. Cell* 9 1081–1091. 10.1091/mbc.9.5.1081 9571241 PMC25331

[B32] McAdams H. H. Arkin A. (1999). It’s a noisy business! Genetic regulation at the nanomolar scale. *Trends Genet.* 15 65–69. 10.1016/S0168-9525(98)01659-X 10098409

[B33] Morano K. A. Grant C. M. Moye-Rowley W. S. (2012). The response to heat shock and oxidative stress in *Saccharomyces cerevisiae*. *Genetics* 190 1157–1195. 10.1534/genetics.111.128033 22209905 PMC3316637

[B34] Murphy K. F. Adams R. M. Wang X. Balazsi G. Collins J. J. (2010). Tuning and controlling gene expression noise in synthetic gene networks. *Nucleic Acids Res.* 38 2712–2726. 10.1093/nar/gkq091 20211838 PMC2860118

[B35] Murphy K. F. Balazsi G. Collins J. J. (2007). Combinatorial promoter design for engineering noisy gene expression. *Proc. Natl. Acad. Sci. U.S.A.* 104 12726–12731. 10.1073/pnas.0608451104 17652177 PMC1931564

[B36] New A. M. Cerulus B. Govers S. K. Perez-Samper G. Zhu B. Boogmans S. (2014). Different levels of catabolite repression optimize growth in stable and variable environments. *PLoS Biol.* 12:e1001764 10.1371/journal.pbio.1001764 PMC3891604 24453942

[B37] Newman J. R. Ghaemmaghami S. Ihmels J. Breslow D. K. Noble M. Derisi J. L. (2006). Single-cell proteomic analysis of *S.* *cerevisiae* reveals the architecture of biological noise. *Nature* 441 840–846. 10.1038/nature04785 16699522

[B38] Octavio L. M. Gedeon K. Maheshri N. (2009). Epigenetic and conventional regulation is distributed among activators of FLO11 allowing tuning of population-level heterogeneity in its expression. *PLoS Genet.* 5:e1000673 10.1371/journal.pgen.1000673 PMC2745563 19798446

[B39] Paliwal S. Iglesias P. A. Campbell K. Hilioti Z. Groisman A. Levchenko A. (2007). MAPK-mediated bimodal gene expression and adaptive gradient sensing in yeast. *Nature* 446 46–51. 10.1038/nature05561 17310144

[B40] Pelet S. Rudolf F. Nadal-Ribelles M. De Nadal E. Posas F. Peter M. (2011). Transient activation of the HOG MAPK pathway regulates bimodal gene expression. *Science* 332 732–735. 10.1126/science.1198851 21551064

[B41] Raser J. M. O’Shea E. K. (2004). Control of stochasticity in eukaryotic gene expression. *Science* 304 1811–1814. 10.1126/science.1098641 15166317 PMC1410811

[B42] Richard M. Yvert G. (2014). How does evolution tune biological noise? *Front. Genet.* 5:374 10.3389/fgene.2014.00374 PMC4211553 25389435

[B43] Rotem E. Loinger A. Ronin I. Levin-Reisman I. Gabay C. Shoresh N. (2010). Regulation of phenotypic variability by a threshold-based mechanism underlies bacterial persistence. *Proc. Natl. Acad. Sci. U.S.A.* 107 12541–12546. 10.1073/pnas.1004333107 20616060 PMC2906590

[B44] Sanchez A. Choubey S. Kondev J. (2013). Regulation of noise in gene expression. *Annu. Rev. Biophys.* 42 469–491. 10.1146/annurev-biophys-083012-130401 23527780

[B45] Sanchez A. Golding I. (2013). Genetic determinants and cellular constraints in noisy gene expression. *Science* 342 1188–1193. 10.1126/science.1242975 24311680 PMC4045091

[B46] Sharon E. Van Dijk D. Kalma Y. Keren L. Manor O. Yakhini Z. (2014). Probing the effect of promoters on noise in gene expression using thousands of designed sequences. *Genome Res.* 24 1698–1706. 10.1101/gr.168773.113 25030889 PMC4199362

[B47] Smith M. C. Sumner E. R. Avery S. V. (2007). Glutathione and Gts1p drive beneficial variability in the cadmium resistances of individual yeast cells. *Mol. Microbiol.* 66 699–712. 10.1111/j.1365-2958.2007.05951.x 17919285 PMC2167119

[B48] To T. L. Maheshri N. (2010). Noise can induce bimodality in positive transcriptional feedback loops without bistability. *Science* 327 1142–1145. 10.1126/science.1178962 20185727

[B49] Venturelli O. S. El-Samad H. Murray R. M. (2012). Synergistic dual positive feedback loops established by molecular sequestration generate robust bimodal response. *Proc. Natl. Acad. Sci. U.S.A.* 109 E3324–E3333. 10.1073/pnas.1211902109 23150580 PMC3511703

[B50] Venturelli O. S. Zuleta I. Murray R. M. El-Samad H. (2015). Population diversification in a yeast metabolic program promotes anticipation of environmental shifts. *PLoS Biol.* 13:e1002042 10.1371/journal.pbio.1002042 PMC4307983 25626086

[B51] Wang J. Atolia E. Hua B. Savir Y. Escalante-Chong R. Springer M. (2015). Natural variation in preparation for nutrient depletion reveals a cost-benefit tradeoff. *PLoS Biol.* 13:e1002041 10.1371/journal.pbio.1002041 PMC4308108 25626068

[B52] Wiseman A. (2005). Avoidance of oxidative-stress perturbation in yeast bioprocesses by proteomic and genomic biostrategies? *Lett. Appl. Microbiol.* 40 37–43. 10.1111/j.1472-765X.2004.01624.x 15613000 PMC7197893

[B53] Zhang Z. Qian W. Zhang J. (2009). Positive selection for elevated gene expression noise in yeast. *Mol. Syst. Biol.* 5:299 10.1038/msb.2009.58 PMC2736655 19690568

