# Supplementary material for: A GRX1 Promoter Variant Confers Constitutive Noisy Bimodal Expression That Increases Oxidative Stress Resistance in Yeast
Source: Front Microbiol. 2018 Sep 19;9:2158. doi: 10.3389/fmicb.2018.02158 (PMC6156533; doi:10.3389/fmicb.2018.02158)
Supplement: Supplementary file 1 [file Data_Sheet_1.PDF]

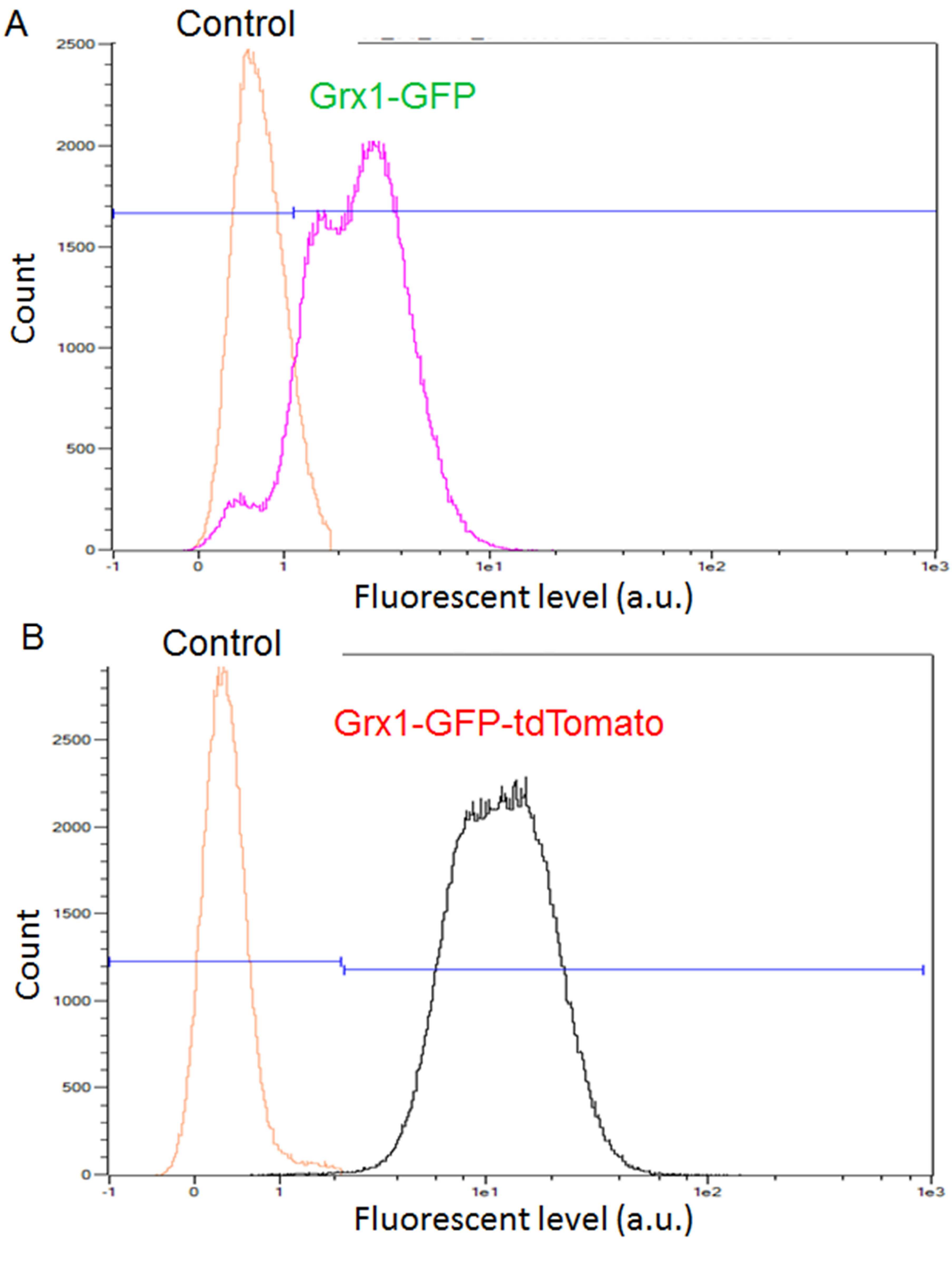


**Supplementary Figure 1.** Fluorescence level conferred by either (A) the C-terminal GFP fusion or (B) the C-terminal double GFP-tdTomato fusion with the *GRX1* ORF compared to the non-fluorescent control strain.
