# Supplementary material for: A GRX1 Promoter Variant Confers Constitutive Noisy Bimodal Expression That Increases Oxidative Stress Resistance in Yeast
Source: Front Microbiol. 2018 Sep 19;9:2158. doi: 10.3389/fmicb.2018.02158 (PMC6156533; doi:10.3389/fmicb.2018.02158)
Supplement: Supplementary file 3 [file Data_Sheet_3.PDF]

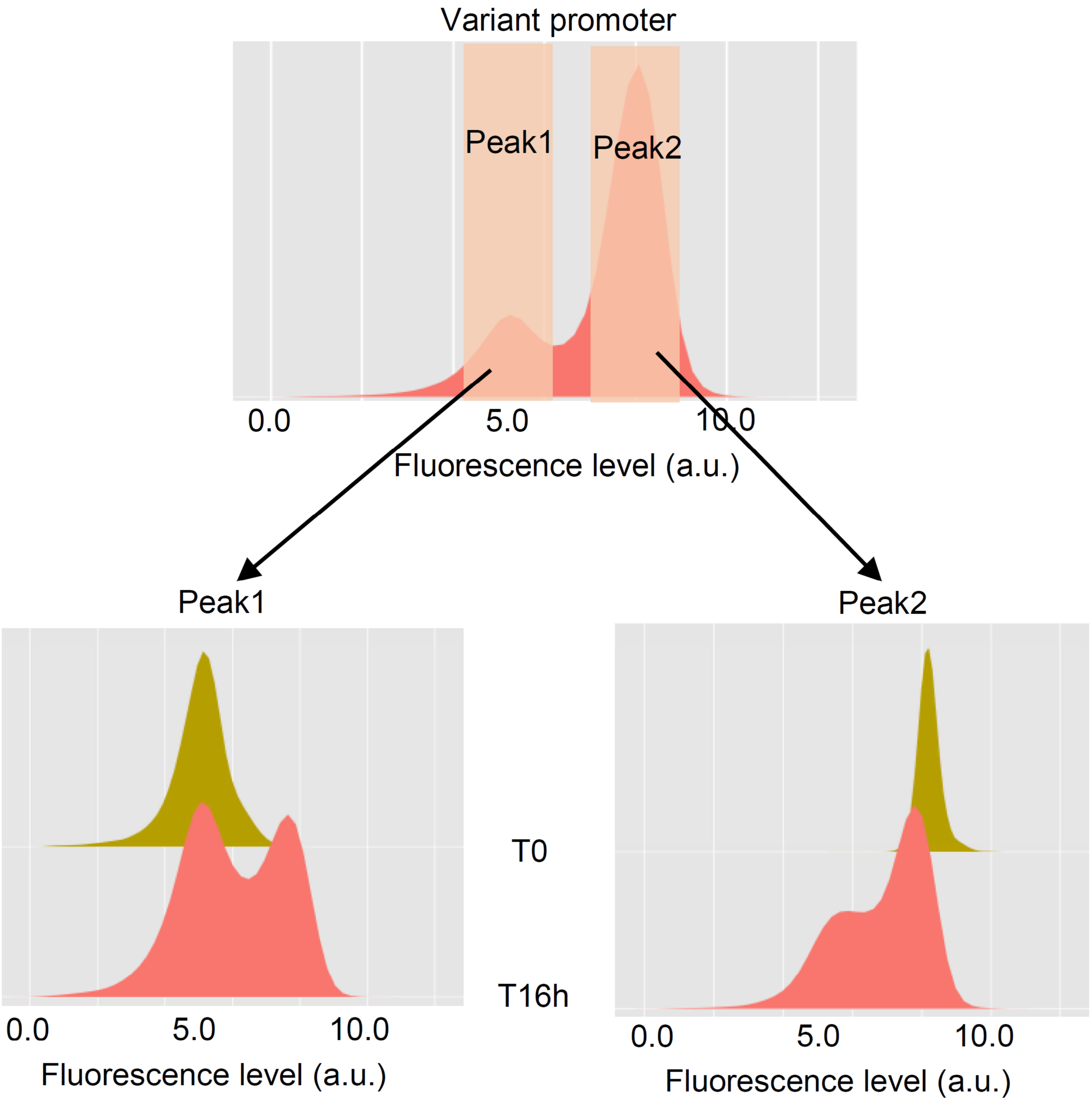

Supplementary Figure 3. Cell sorting of cells around the peak of either the low-expressing cells or the high-expressing cells. At time 0 (T0), each subpopulation indeed present a unimodal expression profile at the expected level. Subsequent growth in non-selective media showed that bimodality was restored in both cases after 16h (T16h).
