# Supplementary material for: A GRX1 Promoter Variant Confers Constitutive Noisy Bimodal Expression That Increases Oxidative Stress Resistance in Yeast
Source: Front Microbiol. 2018 Sep 19;9:2158. doi: 10.3389/fmicb.2018.02158 (PMC6156533; doi:10.3389/fmicb.2018.02158)
Supplement: Supplementary file 4 [file Data_Sheet_4.PDF]

>pGRX1mut
GCCTCCGCCGCTACGGCGGGAACGGCTGCTCTTCGAGTACCTCTGTTTGACTGTTTGACCCAATTGATAAAGACCTGAAT
ACGCCCTCGCATTCTCAAGGTTGTTCTGCGTAATATAGTTTAGCCACGAGATAGCATACGCCGCGCACTTCTTAATAATC
CTAGGCTCATACTTTTCACCGGACCCGCTGTTTTCAGAGTTCGTAGCCATCCCTCTCAGCCTTTGCAGCAGCTTGTCATC
ATTGTACATGACTGTAAACTTCACGCCTTGTGAGATGAACAGATCCAAGAGGTCCAGAGTCCTGGACTGCTGCAGCCGGT
TCCCATACTTTATTTTCTTTCTAAGTGCTCGCGCAGCTTCTTCTTGGTTCACGGTATAGTTATAATCGTTGTGATCTGCT
TTGATAAGCTGAATCAAGGGAGCTAACTCCACTTCTAGCGTATAGTCCCTTGAAGAGACAATTCGAAAGATCGTCTCGGT
GATAGCTGTATGCGGATGATCCGAAAGAAACCCCATGTTCTTGTTTTGCCTGCCTCCAATGCTTTATCACTCTCTCACAC
TGTCACAATCGTGTTGTCTTCATCCTTAGAAAGGATACCACATTGATAAACAACATATATAAAGTTTAACTATTACCTTG
ATCACTTTACACGTCAAGGTCAAAACAGTTTCATAGTTATCACCTTGAAGTATGGCTCCATCATCTATAAAACATGAACT
TCATGCACAAGTGAGCTGTCTACAGATAACGAGCAGCCGCAACGGCGCTTCCAGATTGCGATGCTTGTTTTCTTTATCTA
TTACTGCCTTACTACCCCCCTTGCGCCTCCTGATTCACGTGATGTGGGAATTTTGTCTTGAAAGGAGTAAATATATAAAA
TAAATGAAAAGTTTATATAATATAAAAAGGGACTTTAGCATAAAATAAAGAACTTCGTGCAGTACTTATACGAGCATTCG
CATAATTATACAAATAGACAAAACCTCAGAAGGAAAAAAAATGGTATCTCAAGAAACTATCAAGCACATCAATTGTGTTG
TCTTCATCCTTAGAAAGGATACCACATTGATAAACAACATATATAAAGTTTAACTATTACCTTGATCACTTTACACGTCA
AGGGCAAAACAGTTTCATAGTTATCACCTTGAAGTATGGCTCCATCATCTATAAAACATGAACTTCATGCACAAGTGAGC
TGTCTACAGATAACGAGCAGCCGCAACGGCGTTTCCAGATTGCGATGCTTGTTTTCTTTATCTACTACTGCCTTACTACC
CCCCTTGCGCCTCCTGATTCACGTGATGTGGGAATTTTGTCTTGAAAGGAGTAAATATATAAAATAAATGAAAAGTTTAT
ATAATATAAAAAGGGACTTTAGCATAAAATAA_GAACTTCGTGCAGTACTTATACGAGCATTCGCATAATTATACAAATA
GACAAAACCTCAGAAGGAAAAAAAATGGTATCTCAAGAAACTATCAAGCACGTCAATTGTGTTGTCTTCATCCTTAGAAA
GGATACCACATTGATAAACAACATATATAAAGTTTAACTATTACCTTGATCACTTTACACGTCAAGGTCAAAACAGTTTC
ATAGTTATCACCTTGAAGTATGGCTCCATCATCTATAAAACATGAACTTCATGCACAAGTGAGCTGTCTACAGATAACCA
GCAGCCGCAACGGCGTTTCCAGATTACGATGCTTGTTATCTTTATCTACTACTGCCTTACTACCCCCCTTGCGCCTCCTG
ATTCGCGTGATGTGGGAATTTTGTCTTGAAAGGAGTAAATATATAAAATAAATGAAAAGTTTATATAATATAAAAAGGGA
CTTTAGCATAAAATAAAGAACTTCGTGCAGTACTTATACGAGCATTCGCATAATTATACAAATAGACAAAACCTCAGAAG
GAAAAAAAATGGTATCTCAAGAAACTATCAAGCACGTCAAGGACCTTAT

Supplementary Figure 4. Full sequence of the *GRX1* promoter variant conferring bimodal expression. The mutations in the three repeats are in red and underlined in black, sequences underlined in blue correspond to the primers used for the mutagenic PCR (the reverse primer contains few codons of the beginning of the ORF after ATG), additional nucleotides added at the junction between copies 1 and 2, and between copies 2 and 3 are underlined in yellow.
