# Supplementary material for: A GRX1 Promoter Variant Confers Constitutive Noisy Bimodal Expression That Increases Oxidative Stress Resistance in Yeast
Source: Front Microbiol. 2018 Sep 19;9:2158. doi: 10.3389/fmicb.2018.02158 (PMC6156533; doi:10.3389/fmicb.2018.02158)
Supplement: Supplementary file 7 [file Table_1.DOCX]

| Name |  | Sequences |
| --- | --- | --- |
| C1 | AdtdToma-GFP-for | TGTAACAGCTGCTGGGATTACACATGGCATGGATGAACTATACAAAATTAACATGGTGAGCAAGGG |
| C2 | AdtdToma-rev | AAATGACAAGTTCTTGAAAACAAGAATCTTTTTATTGTCAGTACTTTACTTGTACAGCTCGTCCATG |
| C3 | GRX1-EcoRI-Rev | CCCGGAATTCCCAATTCACCAGTCTCCCTC |
| C4 | GRX1-SalI-For | GGGTGTCGACAGCTCTCTTCCTCTGAGGACTC |
| C5 | rev-mut | GTGCTTGATAGTTTCTTGAGATACC |
| C6 | SS-for-mut | GTGTTGTCTTCATCCTTAGAAAGG |
| C7 | Ps-GRX1-URA-For | GGCGGGAACGGCTGCTCTTCGAGTACCTCTGTTTGACTGTTTGACCCGTTTATCGCTGGCAAC |
| C8 | Ps-GRX1-URA-Rev | TCTGCAATAAGGTCCTTGACGTGCTTGATAGTTTCTTGAGATACCGGCTGGCCTGTTGAACAAG |

**Supplementary Table 1.** List of primers used in this study.
