# Supplementary material for: A GRX1 Promoter Variant Confers Constitutive Noisy Bimodal Expression That Increases Oxidative Stress Resistance in Yeast
Source: Front Microbiol. 2018 Sep 19;9:2158. doi: 10.3389/fmicb.2018.02158 (PMC6156533; doi:10.3389/fmicb.2018.02158)
Supplement: Supplementary file 8 [file Table_2.DOCX]

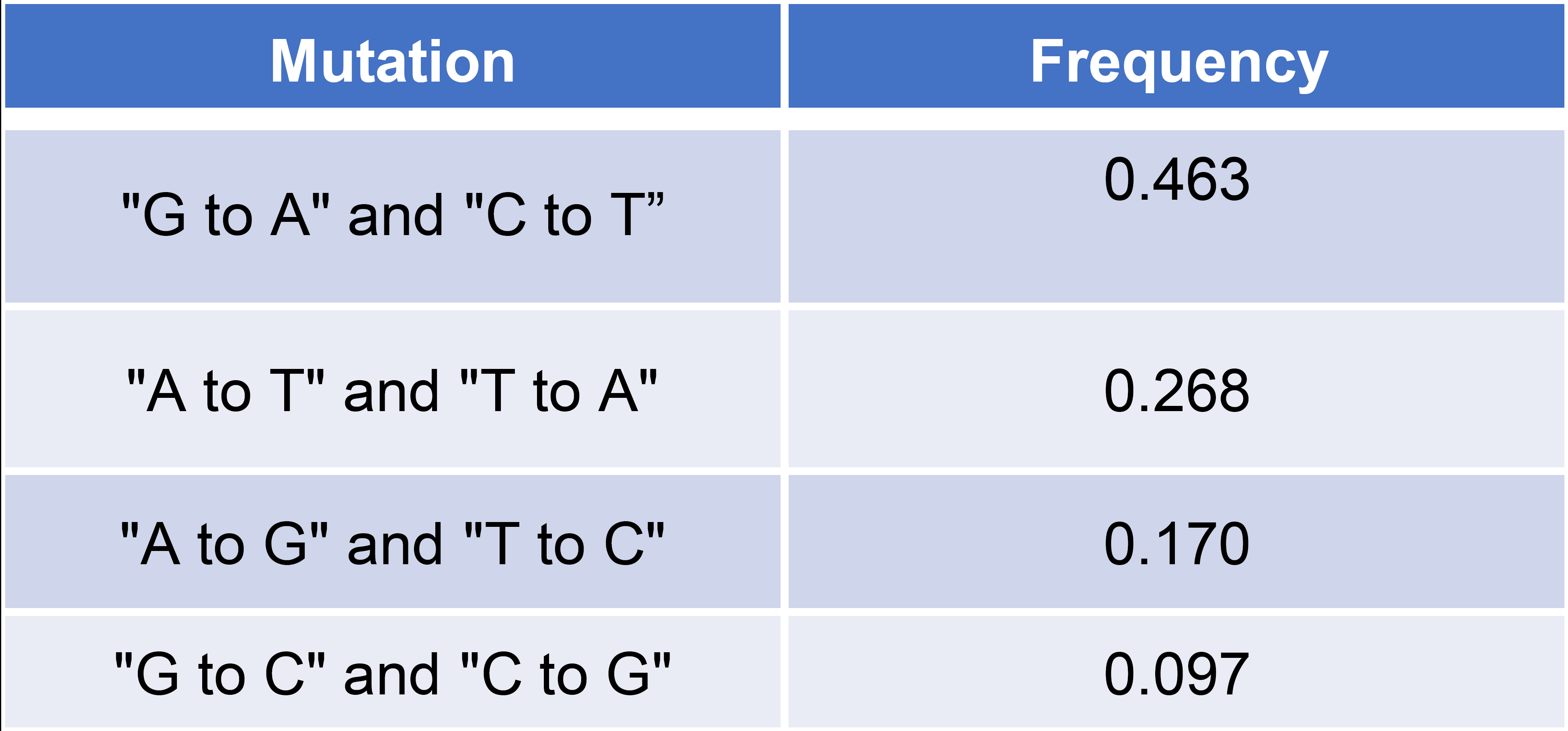


**Supplementary Table 2.** Mutation frequencies among the *GRX1* promoter variants produced by error-prone PCR.
