# Supplementary material for: A GRX1 Promoter Variant Confers Constitutive Noisy Bimodal Expression That Increases Oxidative Stress Resistance in Yeast
Source: Front Microbiol. 2018 Sep 19;9:2158. doi: 10.3389/fmicb.2018.02158 (PMC6156533; doi:10.3389/fmicb.2018.02158)
Supplement: Supplementary file 9 [file Table_3.DOCX]

| **Clones from mutation library** | | |  |  |
| --- | --- | --- | --- | --- |
| Name | Fluorescence mean | Fluorescence noise | sd of Fluorescence mean | sd Fluorescence noise |
| M1 | 6,7207 | 0,0177 | 0,4534 | 0,0125 |
| M2 | 7,8623 | 0,0095 | 0,5326 | 0,0072 |
| M3 | 7,8271 | 0,0099 | 0,6849 | 0,0060 |
| M4 | 8,0214 | 0,0087 | 0,6573 | 0,0039 |
| M5 | 7,6416 | 0,0115 | 0,9000 | 0,0074 |
| M6 | 7,5780 | 0,0135 | 1,0353 | 0,0100 |
| M7 | 7,9218 | 0,0100 | 0,4061 | 0,0062 |
| M8 | 7,5814 | 0,0122 | 0,8377 | 0,0079 |
| M9 | 7,3040 | 0,0127 | 0,5177 | 0,0064 |
| M10 | 7,7535 | 0,0121 | 0,6816 | 0,0092 |
| M11 | 7,8046 | 0,0130 | 0,6390 | 0,0089 |
| M12 | 7,7751 | 0,0140 | 0,5458 | 0,0077 |
| M13 | 8,1957 | 0,0129 | 0,4767 | 0,0126 |
| M14 | 7,8001 | 0,0109 | 0,5993 | 0,0073 |
| M15 | 8,1500 | 0,0104 | 0,5836 | 0,0073 |
| M16 | 8,1838 | 0,0099 | 0,2570 | 0,0065 |
| M17 | 7,3512 | 0,0186 | 0,7969 | 0,0132 |
| M18 | 7,3356 | 0,0184 | 0,7993 | 0,0123 |
| M19 | 7,2948 | 0,0162 | 0,1252 | 0,0048 |
| M21 | 7,6879 | 0,0137 | 0,7273 | 0,0085 |
| M22 | 7,5423 | 0,0146 | 0,7522 | 0,0103 |
| M23 | 8,1609 | 0,0098 | 0,4679 | 0,0065 |
| M24 | 7,6475 | 0,0114 | 0,7421 | 0,0072 |
| M25 | 8,2471 | 0,0383 | 0,7062 | 0,0165 |
| M26 | 7,9281 | 0,0112 | 0,5575 | 0,0078 |
| M27 | 7,9515 | 0,0132 | 0,5008 | 0,0123 |
| M28 | 7,9553 | 0,0123 | 0,4922 | 0,0081 |
| M29 | 7,9282 | 0,0106 | 0,4668 | 0,0066 |
| M30 | 8,1048 | 0,0082 | 0,3367 | 0,0051 |
| M31 | 7,5021 | 0,0113 | 0,7262 | 0,0077 |
| M32 | 7,7662 | 0,0111 | 0,9493 | 0,0080 |
| M33 | 7,4351 | 0,0160 | 0,7866 | 0,0091 |
| M34 | 6,8101 | 0,0165 | 0,7567 | 0,0108 |
| M35 | 7,6789 | 0,0119 | 0,7920 | 0,0072 |
| M36 | 7,3641 | 0,0133 | 0,9443 | 0,0082 |
| M37 | 7,1245 | 0,0133 | 0,8582 | 0,0069 |
| M38 | 7,1636 | 0,0110 | 0,7583 | 0,0047 |
| M39 | 7,9957 | 0,0091 | 0,5398 | 0,0092 |
| M40 | 7,6935 | 0,0101 | 0,4969 | 0,0070 |
| M41 | 8,1444 | 0,0071 | 0,2648 | 0,0037 |
| M42 | 8,0551 | 0,0064 | 0,0704 | 0,0028 |
| M43 | 8,2462 | 0,0085 | 0,2731 | 0,0054 |
| M44 | 8,0730 | 0,0107 | 0,3571 | 0,0070 |
| M45 | 7,7401 | 0,0165 | 0,5257 | 0,0112 |
| M46 | 7,9251 | 0,0119 | 0,5967 | 0,0079 |
| M47 | 7,7465 | 0,0117 | 0,6775 | 0,0079 |
| M48 | 7,8413 | 0,0134 | 0,6018 | 0,0089 |
| M49 | 7,7417 | 0,0105 | 0,6682 | 0,0069 |
| M50 | 7,8618 | 0,0085 | 0,3736 | 0,0045 |
| M51 | 8,5136 | 0,0101 | 0,3291 | 0,0096 |
| M52 | 7,9620 | 0,0120 | 0,3564 | 0,0068 |
| M53 | 7,9241 | 0,0119 | 0,3977 | 0,0070 |
| M54 | 8,0181 | 0,0097 | 0,1842 | 0,0050 |
| M55 | 7,8224 | 0,0138 | 0,7278 | 0,0098 |
| M56 | 7,7900 | 0,0142 | 0,8309 | 0,0093 |
| M57 | 8,0251 | 0,0140 | 0,4197 | 0,0096 |
| M58 | 7,5509 | 0,0173 | 0,7089 | 0,0114 |
| M59 | 7,4603 | 0,0145 | 0,7136 | 0,0093 |
| M60 | 7,0919 | 0,0122 | 0,8800 | 0,0050 |
| M61 | 7,5229 | 0,0114 | 0,8625 | 0,0084 |
| M62 | 7,8629 | 0,0110 | 0,2195 | 0,0040 |
| M63 | 8,1081 | 0,0070 | 0,1602 | 0,0037 |
| M64 | 8,0119 | 0,0104 | 0,4576 | 0,0059 |
| M65 | 8,3282 | 0,0111 | 0,2952 | 0,0045 |
| M66 | 8,2345 | 0,0093 | 0,1791 | 0,0052 |
| M67 | 7,7004 | 0,0117 | 0,5856 | 0,0068 |
| M68 | 8,0625 | 0,0143 | 0,4659 | 0,0095 |
| M69 | 8,0423 | 0,0125 | 0,4156 | 0,0087 |
| M70 | 7,8803 | 0,0096 | 0,5823 | 0,0062 |
| M71 | 7,8236 | 0,0111 | 0,5343 | 0,0072 |
| M72 | 8,2097 | 0,0105 | 0,2169 | 0,0071 |
| M73 | 7,9587 | 0,0120 | 0,6623 | 0,0082 |
| M74 | 7,9708 | 0,0127 | 0,5437 | 0,0078 |
| M75 | 8,2454 | 0,0090 | 0,2159 | 0,0059 |
| M76 | 8,1374 | 0,0080 | 0,5178 | 0,0050 |
| M77 | 8,1183 | 0,0089 | 0,3070 | 0,0053 |
| M78 | 8,2001 | 0,0071 | 0,2075 | 0,0037 |
| M79 | 8,0151 | 0,0109 | 0,3489 | 0,0072 |
| M80 | 7,5959 | 0,0170 | 0,4872 | 0,0110 |
| M81 | 7,5002 | 0,0185 | 0,7408 | 0,0132 |
| M82 | 7,7841 | 0,0166 | 0,5296 | 0,0124 |
| M83 | 7,7745 | 0,0106 | 0,6519 | 0,0053 |
| M84 | 8,1281 | 0,0111 | 0,3273 | 0,0077 |
| M85 | 7,7634 | 0,0114 | 0,7484 | 0,0073 |
| M86 | 7,8946 | 0,0093 | 0,8331 | 0,0059 |
| M87 | 8,5762 | 0,0060 | 0,1704 | 0,0040 |
| M88 | 8,0540 | 0,0092 | 0,3615 | 0,0068 |
| M89 | 8,1836 | 0,0060 | 0,6989 | 0,0030 |
| M90 | 7,9602 | 0,0079 | 0,3201 | 0,0043 |
| M91 | 7,7573 | 0,0087 | 0,4944 | 0,0075 |
| M92 | 7,9561 | 0,0139 | 0,3972 | 0,0105 |
| M93 | 7,8665 | 0,0158 | 0,5086 | 0,0110 |
| M94 | 7,7820 | 0,0087 | 0,9340 | 0,0077 |
| M95 | 7,7690 | 0,0156 | 0,6785 | 0,0094 |
| M96 | 8,1242 | 0,0104 | 0,4013 | 0,0094 |
| M97 | 7,4714 | 0,0159 | 0,3527 | 0,0063 |
| M98 | 7,5551 | 0,0140 | 0,6913 | 0,0029 |
| M99 | 8,1518 | 0,0033 | 0,1049 | 0,0007 |
| M100 | 7,9944 | 0,0089 | 0,3417 | 0,0056 |
| M101 | 8,0590 | 0,0088 | 0,3509 | 0,0052 |
| M102 | 7,2057 | 0,0084 | 0,6062 | 0,0040 |
| M103 | 7,9446 | 0,0083 | 0,4519 | 0,0050 |
| M104 | 7,9712 | 0,0070 | 0,1065 | 0,0038 |
| M105 | 8,0267 | 0,0082 | 0,3090 | 0,0054 |
| M106 | 7,9915 | 0,0078 | 0,3147 | 0,0049 |
| M107 | 8,1376 | 0,0086 | 0,4881 | 0,0051 |
| M108 | 7,9789 | 0,0088 | 0,3376 | 0,0054 |
| M109 | 8,0463 | 0,0095 | 0,4238 | 0,0063 |
| M110 | 8,1100 | 0,0073 | 0,4561 | 0,0055 |
| M111 | 8,4599 | 0,0043 | 0,1050 | 0,0016 |
| M112 | 7,8930 | 0,0095 | 0,6481 | 0,0066 |
| M113 | 7,9182 | 0,0104 | 0,5559 | 0,0059 |
| M114 | 8,2068 | 0,0064 | 0,4531 | 0,0033 |
| M115 | 8,0790 | 0,0104 | 0,2941 | 0,0063 |
| M116 | 7,7858 | 0,0156 | 0,4740 | 0,0120 |
| M117 | 7,8774 | 0,0130 | 0,5249 | 0,0090 |
| M118 | 8,0292 | 0,0132 | 0,4356 | 0,0092 |
| M119 | 7,9425 | 0,0102 | 0,5523 | 0,0064 |
| M120 | 7,7833 | 0,0093 | 0,7651 | 0,0050 |
| M121 | 7,7407 | 0,0116 | 0,4611 | 0,0070 |
| M122 | 8,0644 | 0,0071 | 0,7086 | 0,0041 |
| M123 | 8,5850 | 0,0058 | 0,2046 | 0,0030 |
| M124 | 7,8250 | 0,0086 | 0,6081 | 0,0058 |
| M125 | 7,7840 | 0,0092 | 0,6255 | 0,0052 |
| M126 | 8,1404 | 0,0079 | 0,3442 | 0,0050 |
| M127 | 8,1395 | 0,0125 | 0,2091 | 0,0065 |
| M128 | 7,5858 | 0,0153 | 0,6759 | 0,0100 |
| M129 | 7,7694 | 0,0157 | 0,5399 | 0,0108 |
| M130 | 7,3443 | 0,0164 | 0,4911 | 0,0114 |
| M131 | 7,7577 | 0,0127 | 0,5524 | 0,0074 |
| M132 | 7,2222 | 0,0122 | 0,7326 | 0,0068 |
| M133 | 7,3953 | 0,0105 | 0,7030 | 0,0066 |
| M134 | 7,9968 | 0,0093 | 0,5407 | 0,0057 |
| M135 | 8,4030 | 0,0036 | 0,0505 | 0,0009 |
| M136 | 7,9024 | 0,0071 | 0,5666 | 0,0043 |
| M137 | 8,1816 | 0,0089 | 0,6657 | 0,0057 |
| M138 | 8,0215 | 0,0077 | 0,4264 | 0,0044 |
| M139 | 8,1234 | 0,0098 | 0,2486 | 0,0063 |
| M140 | 7,8912 | 0,0089 | 0,3330 | 0,0058 |
| M141 | 7,8272 | 0,0106 | 0,5653 | 0,0075 |
| M142 | 7,7055 | 0,0095 | 0,3436 | 0,0056 |
| M143 | 7,9365 | 0,0075 | 0,4981 | 0,0045 |
| M144 | 7,7207 | 0,0085 | 0,3946 | 0,0045 |
| M145 | 7,8080 | 0,0097 | 0,4463 | 0,0059 |
| M146 | 8,0067 | 0,0094 | 0,4727 | 0,0060 |
| M147 | 8,5228 | 0,0054 | 0,2493 | 0,0007 |
| M149 | 7,5068 | 0,0064 | 0,3627 | 0,0029 |
| M150 | 7,5443 | 0,0123 | 0,1728 | 0,0042 |
| M151 | 8,1413 | 0,0091 | 0,2639 | 0,0045 |
| M152 | 8,0832 | 0,0122 | 0,4255 | 0,0070 |
| M153 | 7,5561 | 0,0204 | 0,2442 | 0,0089 |
| M154 | 7,5331 | 0,0182 | 0,3042 | 0,0092 |
| M155 | 7,3163 | 0,0166 | 0,2398 | 0,0062 |
| M156 | 7,7473 | 0,0126 | 0,5029 | 0,0074 |
| M157 | 7,2423 | 0,0117 | 0,6306 | 0,0050 |
| M158 | 8,0691 | 0,0086 | 0,3547 | 0,0052 |
| M159 | 7,9676 | 0,0111 | 0,3525 | 0,0070 |
| M160 | 8,5406 | 0,0038 | 0,0944 | 0,0006 |
| M161 | 7,7031 | 0,0118 | 0,2916 | 0,0053 |
| M162 | 7,9958 | 0,0092 | 0,4406 | 0,0056 |
| M163 | 7,9433 | 0,0095 | 0,2523 | 0,0052 |
| M164 | 7,9917 | 0,0120 | 0,4075 | 0,0071 |
| M165 | 7,7288 | 0,0166 | 0,4833 | 0,0120 |
| M166 | 7,5955 | 0,0186 | 0,1769 | 0,0068 |
| M167 | 7,4918 | 0,0157 | 0,3930 | 0,0087 |
| M168 | 7,6457 | 0,0153 | 0,4616 | 0,0051 |
| M169 | 7,5826 | 0,0108 | 0,6651 | 0,0070 |
| M170 | 7,9836 | 0,0112 | 0,4317 | 0,0068 |
| M171 | 7,7380 | 0,0107 | 0,6139 | 0,0057 |
| M172 | 8,7085 | 0,0030 | 0,1030 | 0,0005 |
| M173 | 8,2058 | 0,0091 | 0,2913 | 0,0057 |
| M174 | 7,9774 | 0,0087 | 0,3576 | 0,0053 |
| M175 | 8,0083 | 0,0084 | 0,3277 | 0,0044 |
| M176 | 7,9585 | 0,0050 | 0,1169 | 0,0026 |
| M177 | 7,9492 | 0,0151 | 0,4282 | 0,0103 |
| M178 | 7,8240 | 0,0148 | 0,4889 | 0,0108 |
| M179 | 8,1431 | 0,0111 | 0,3799 | 0,0072 |
| M180 | 7,6727 | 0,0110 | 0,4569 | 0,0070 |
| M181 | 7,7098 | 0,0075 | 0,6446 | 0,0041 |
| M182 | 7,8724 | 0,0072 | 0,3152 | 0,0037 |
| M183 | 8,0490 | 0,0093 | 0,4754 | 0,0058 |
| M184 | 8,5661 | 0,0041 | 0,1685 | 0,0008 |
| M185 | 8,3438 | 0,0068 | 0,2882 | 0,0053 |
| M186 | 8,5600 | 0,0084 | 0,1306 | 0,0056 |
| M187 | 8,2547 | 0,0089 | 0,2613 | 0,0051 |
| M188 | 8,6415 | 0,0052 | 0,0707 | 0,0009 |
| M189 | 8,4361 | 0,0054 | 0,1085 | 0,0018 |
| M190 | 8,1643 | 0,0098 | 0,4205 | 0,0077 |
| M191 | 7,4728 | 0,0123 | 0,3097 | 0,0076 |
| M192 | 8,1896 | 0,0113 | 0,2067 | 0,0059 |
| M193 | 8,1292 | 0,0116 | 0,2237 | 0,0071 |
| M194 | 8,3036 | 0,0095 | 0,3358 | 0,0049 |
| M195 | 8,2319 | 0,0080 | 0,2732 | 0,0053 |

**Supplementary Table 3.** Mean and noise levels of about 200 clones from the *GRX1* promoter variants library. Results are the mean of 3 independent experiments and standard deviations are provided for both the mean and noise levels. The outlier clone M25 is highlighted in red.
